# Loss of MBD2 attenuates MLL-AF9-driven leukemogenesis by suppressing the leukemic cell cycle via CDKN1C

**DOI:** 10.1038/s41389-021-00366-3

**Published:** 2021-11-17

**Authors:** Kuangguo Zhou, Mi Zhou, Ling Cheng, Xing Chen, Xiaomin Wang, Yajing Chu, Qilin Yu, Shu Zhang, Na Wang, Lei Zhao, Di Wang, Liang Huang, Congyi Wang, Weiping Yuan, Jianfeng Zhou

**Affiliations:** 1 Department of Hematology, Tongji Hospital, Tongji Medical College, Huazhong University of Science and Technology, Wuhan, Hubei China; 2 Department of Geriatrics, Tongji Hospital, Tongji Medical College, Huazhong University of Science and Technology, Wuhan, Hubei China; 3 State Key Laboratory of Experimental Hematology, National Clinical Research Center for Blood Diseases, Institute of Hematology and Blood Diseases Hospital, Chinese Academy of Medical Sciences and Peking Union Medical College, Tianjin, China; 4 The Center for Biomedical Research, Tongji Hospital, Tongji Medical College, Huazhong University of Science and Technology, Wuhan, China; 5 Cancer Biology Research Center, Tongji Hospital, Tongji Medical College, Huazhong University of Science and Technology, Wuhan, Hubei China

**Keywords:** Acute myeloid leukaemia, Targeted therapies

## Abstract

Acute myeloid leukemia (AML) is a deadly cancer characterized by an expanded self-renewal capacity that is associated with the accumulation of immature myeloid cells. Emerging evidence shows that methyl-CpG-binding domain protein 2 (MBD2), a DNA methylation reader, often participates in the transcriptional silencing of hypermethylated genes in cancer cells. Nevertheless, the role of MBD2 in AML remains unclear. Herein, by using an MLL-AF9 murine model and a human AML cell line, we observed that loss of MBD2 could delay the initiation and progression of leukemia. MBD2 depletion significantly reduced the leukemia burden by decreasing the proportion of leukemic stem cells (LSCs) and inhibiting leukemia cell proliferation in serial transplantation experiments, thereby allowing leukemic blasts to transition to a more mature state reflecting normal myelopoiesis. Both gene expression analyses and bioinformatic studies revealed that MBD2 negatively modulated genes related to myeloid differentiation, and was necessary to sustain the MLL-AF9 oncogene-induced gene program. We further demonstrated that MBD2 could promote LSC cell cycle progression through epigenetic regulation of *CDKN1C* transcription probably by binding to its promoter region. Taken together, our data suggest that MBD2 promotes AML development and could be a therapeutic target for myeloid malignancies.

## Introduction

Acute myeloid leukemia (AML) is a deadly cancer characterized by an expanded self-renewal capacity linked with the accumulation of immature myeloid cells. The 5-year overall survival rate for adult AML is only 30–40% using current treatments and has only slightly improved in the past 30 years [1]. Leukemic cells with stem cell properties, called leukemic stem cells (LSCs), are believed to contribute to the maintenance and recurrence of leukemia [2]. Self-renewal and differentiation blockade are two key features of LSCs [3]. The strategies for targeting LSCs have yet to be improved, while the induction of myeloid leukemia differentiation has been used as a treatment approach [4, 5]. To improve the treatment of AML, concerted efforts have been made to delineate regulatory events that can either promote the differentiation and/or reduce the proliferation of LSCs.

Several studies have indicated that cancer cells have been reprogrammed to non-tumoral fates by epigenetic therapeutic protocols. Leukemic transformation has also been shown to be a process of epigenetic reprogramming [6]. Inhibition of epigenetic regulatory factors is a possible therapeutic method against oncogenic transcription. Aberrant DNA methylation has been shown in a variety of hematologic malignancies, such as myelodysplastic syndromes and AML. However, growing evidence suggests that the lack of specificity of treatment with a nucleotide analog, DNA methyltransferase inhibitors, might cause off-target effects and certain toxicities.

To circumvent these limitations, targeting the readers of DNA methylation may serve as a suitable alternative for precision epi-therapies. Methyl-CpG-binding domain protein 2 (MBD2) has been proposed as the member that has the greatest effect on gene silencing in the MBD family and is also the only MBD protein that is highly expressed in the spleen [7]. MBD2 also has cell- and tissue-specific functions that are context-dependent [8]. Many studies have demonstrated that MBD2 might bind to hypermethylated promoters of tumor suppressor genes and contribute to their transcriptional silencing in multiple tumors, such as ovarian cancer [9], renal cell carcinoma [10], and hepatocellular carcinoma [11]. Our previous study also showed that MBD2 acts as a tumor suppressor in T cell acute lymphoblastic leukemia (T-ALL) lymphomagenesis in murine experiments [12]. However, little is known about how MBD2 functionally contributes to AML pathogenesis.

Numerous studies have used MLL-rearranged leukemia models to investigate the development and, particularly, the epigenetic program of leukemia cells [13]. According to the Oncomine database analysis by Metzeler et al., the MBD2 expression level in French–American–British (FAB) subtype M4/M5 AML cells is higher than that in granulocytes or monocytes from healthy donors or among cells of other subtypes of AML [14]. It is worth noting that MLL rearrangement is the most often observed rearrangement in M4/M5 AML, indicating that MBD2 may be involved in the pathogenesis of MLL-rearranged AML. Therefore, we hypothesize that MBD2 might functionally contribute to MLL-rearranged AML pathogenesis.

Herein, we genetically inactivated MBD2 in a mouse model of AML induced by MLL-AF9 to study the role of MBD2 in AML. Intriguingly, we found that MBD2 depletion reduced colony formation in leukemia cells, increased differentiation, and delayed leukemogenesis by arresting the cell cycle of LSCs. By using an MLL-AF9 murine model and a human AML cell line, we showed that MBD2 promotes LSC proliferation in part through silencing of the tumor suppressor CDKN1C in AML patients.

## Results

### High levels of MBD2 expression are related to poor clinical outcomes in AML patients

**Fig. 1:**
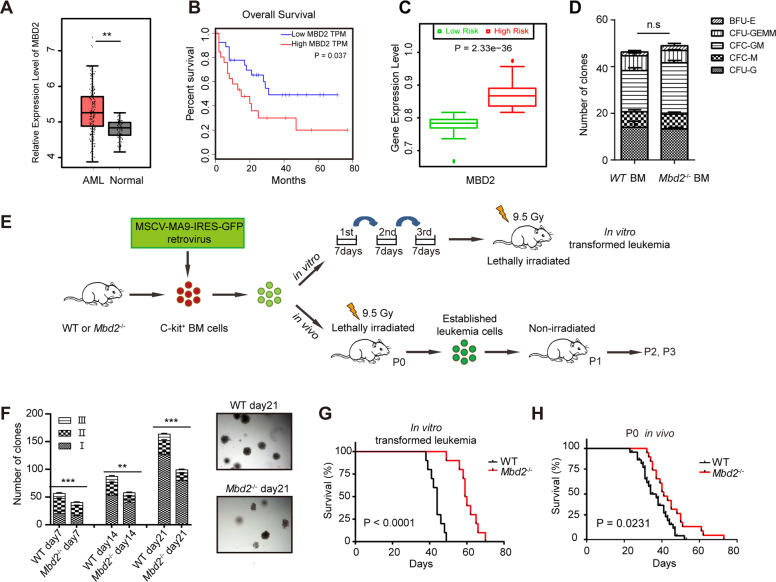
MBD2 is associated with adverse survival outcomes in AML patients and plays an important role in the transformation of MLL-AF9 leukemia. **A** In silico analysis of MBD2 expression in AML patients (*n* = 173) and in the normal population (*n* = 70) (from TCGA and GTEx database through GEPIA website). **B** Kaplan–Meier analysis of MBD2 expression in AML patients from TCGA database through the GEPIA website. High MBD2 TPM, patients with MBD2 levels in the top 25% of values (*n* = 27); low MBD2 TPM, patients with MBD2 levels in the bottom 25% of values (*n* = 27). TPM transcripts per million. **C** Box plots show MBD2 expression levels and *P* values obtained by the *t* test in high-risk (red) and low-risk (green) groups of AML patients. Error bars, s.d. **D** Bar graphs show colony formation assay of *Mbd2*^−/−^ healthy BM cells compared to WT healthy BM cells. Fourteen days after plating, colony differentiation was scored. Error bars, s.d. Colonies were defined as follows: burst-forming unit erythroid (BFU-E), colony-forming unit monocyte (CFU-M), granulocyte–macrophage (CFU-GM), and granulocyte-erythroid-macrophage megakaryocyte (CFU-GEMM). **E** Experimental design of the *Mbd2*^−/−^ or WT murine MLL-AF9-induced AML models. **F** Bar graphs (left) show the colony-forming capacity of *Mbd2*^−^^/−^ MLL-AF9-transformed cells compared to WT MLL-AF9 cells; the colonies were counted after 7 days of culture. Type I, compact colonies included mostly immature myeloid precursors; type II, colonies had a compact center and a halo of loose cells, and type III, colonies had dispersed cells but no center. Colonies of type I to type II to type III reflected the progressive loss of stemness in AML cells. Colony-forming units (CFUs) per 2000 cells averaged from two independent experiments are represented as the mean ± s.d. of triplicates. Representative leukemia colonies observed under an inverted microscope are depicted (right). **G** Kaplan–Meier analysis of recipient mice transplanted with in vitro MLL-AF9-transformed *Mbd2*^−/−^ and WT cells (*n* ≥ 10 per group, two independent experiments). **H** Kaplan–Meier analysis of P0 recipients in the *Mbd2*^−/−^ and WT groups (*n* ≥ 10 per group, two independent experiments). n.s, no significance. **P < 0.005, and ***P < 0.001.

To explore the potential clinical relevance of MBD2 expression in AML patients and in the normal population, we searched the GEPIA website and found that MBD2 was overexpressed in several types of cancer, such as AML (Fig. 1A and Supplementary Fig. S1). High levels of MBD2 expression were correlated with poor clinical outcomes in AML patients (Fig. 1B, C). Our previous study showed that MBD2 was not required for normal hematopoiesis [12]. Furthermore, we found no difference in the colony-forming ability of myeloid progenitors between the *Mbd2*^−/−^ and wild-type (WT) healthy mice (Fig. 1D).

### MBD2 deletion delays MLL-AF9-induced leukemogenesis

To examine the role of MBD2 in AML initiation and maintenance, we generated an MLL-AF9-induced mouse AML model (Fig. 1E). The expression of MBD2 messenger RNA (mRNA) and protein was absent in *Mbd2*^−/−^ mice but present in WT littermates (Supplementary Fig. S2A). We did not observe significant differences in the expression of other members of the MBD family (Supplementary Fig. S2B). To investigate the effect of MBD2 deletion on leukemia transformation, colony formation assays were used to evaluate the self-renewal capacity of WT and *Mbd2*^−/−^ MLL-AF9 mice in vitro. *Mbd2*^−/−^ colonies were smaller and more diffuse than WT colonies (Fig. 1F and Supplementary Fig. S2C), supporting the role of MBD2 in promoting leukemia transformation [15, 16].

Next, we examined whether MBD2 deletion could attenuate MLL-AF9-driven leukemogenesis in vivo. *Mbd2*^−/−^ and WT MLL-AF9-transformed cells obtained from the third generation of colonies were transplanted into mice irradiated with sublethal doses to induce leukemia. Recipient mice from both the *Mbd2*^−/−^ group and the WT group developed AML, and the GFP^+^ cells were shown to be B220^−^CD3^−^Mac-1^+^ by flow cytometric analysis, suggesting the construction of a myeloid leukemia model (Supplementary Fig. S2D). The latent period of recipient mice with MLL-AF9-transformed *Mbd2*^−/−^ cells was significantly longer than that of the WT mice, indicating that MBD2 deficiency delayed leukemia onset (Fig. 1G, H). Together, these data suggest that MBD2 deletion significantly impairs the colony-forming capacity of MLL-AF9-transformed cells and delays MLL-AF9-driven leukemia initiation.

### Loss of MBD2 suppresses AML progression and reduces the leukemic burden

**Fig. 2:**
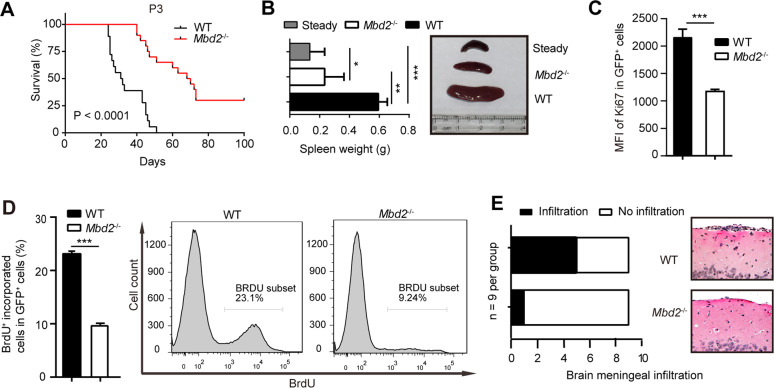
Knockout of MBD2 attenuates AML proliferation and infiltration. **A** Kaplan–Meier survival curves for P3 mice (*n* ≥ 10 per group, two independent experiments). **B** Weights of the spleens (left) and a representative picture of spleens (right) obtained from leukemic mice are shown. ‘Steady’ indicates the spleen from a corresponding healthy mouse. Error bars, s.d. **C** Flow cytometric determination of Ki67 proliferation indexes reported as the mean fluorescence intensities (MFIs). Error bars, s.d. **D** BrdU^+^ cells among the total GFP^+^ leukemia cells (left panel) and representative histograms (right panel) of *Mbd2*^−/−^ versus WT leukemic mice. The data are shown as the mean percentage ± s.d., *n* = 5 per group, 2 independent experiments. **E** The numbers of WT or *Mbd2*^−/−^ leukemic mice (*n* = 9 per group, two independent experiments) with meningeal leukemia (left panel). Representative hematoxylin and eosin staining (HE, right panel) images (×100) of infiltration of AML cells in the brain meningeal spaces of WT and *Mbd2*^−/−^ leukemic mice. Scale bar, 10 μm. *P < 0.05, **P < 0.005, and ***P < 0.001.

To investigate the functional impact of MBD2 deletion on the development of MLL-AF9-driven leukemia, we generated a serial transplantation mouse model. Through the subsequent rounds of transplantation, MBD2 deletion attenuated the proliferation of leukemic cells and extended the lifespan of MLL-rearranged mice compared to that of the control P1–P3 recipients (Fig. 2A and Supplementary Fig. S3A, B).

To determine the malignant characteristics of leukemia cells after MBD2 deletion, cell cycle distribution, proliferation, apoptosis, and meningeal leukemia infiltration were compared between the *Mbd2*^−/−^ and WT groups. The *Mbd2*^−/−^ P3 recipient mice survived much longer than the P2 recipient mice, and the following experiments were carried out with P3 mouse cells. Compared with the WT conditions, the loss of MBD2 significantly decreased the number of white blood cells (WBCs) and leukemic cell infiltration in the peripheral blood, which was consistent with the reduced leukemia burden in *Mbd2*^*−*/−^ AML mice (Supplementary Fig. S3C). In the *Mbd2*^*−*/−^ group, splenomegaly in MLL-AF9 leukemic mice was relieved, as indicated by a decreased spleen weight (mg) to body weight (g) ratio and a remarkably decreased spleen size compared with that in the WT group (Fig. 2B). Cell cycle analysis showed an overt decrease in the proportion of actively cycling cells according to Ki67 staining (Fig. 2C). Bromodeoxyuridine (BrdU) incorporation studies in vivo showed that *Mbd2*^−/−^ AML cells propagated much more slowly than WT cells (Fig. 2D). We found no significant difference in apoptosis between *Mbd2*^−/−^ and WT AML cells (Supplementary Fig. S3D). We also performed homing assays to confirm that the homing potential of leukemia cells was not affected after MBD2 deletion (Supplementary Fig. S3E). Strikingly, *Mbd2*^*−*/−^ AML cells were much less aggressive than WT AML cells according to liquid culture and colony formation assays in vitro, which were applied to investigate the dynamic effect and colony-forming capacity of leukemia cells (Supplementary Fig. S3F, G). Amazingly, the WT AML mice presented with meningeal leukemia infiltration much more frequently than *Mbd2*^*−*/−^ AML mice (Fig. 2E). Therefore, loss of MBD2 significantly impaired AML progression in vivo and extended the lifespan of leukemic mice.

### MBD2 deletion impairs LSC function, leads to G0/G1 arrest, and induces the differentiation of leukemic cells

**Fig. 3:**
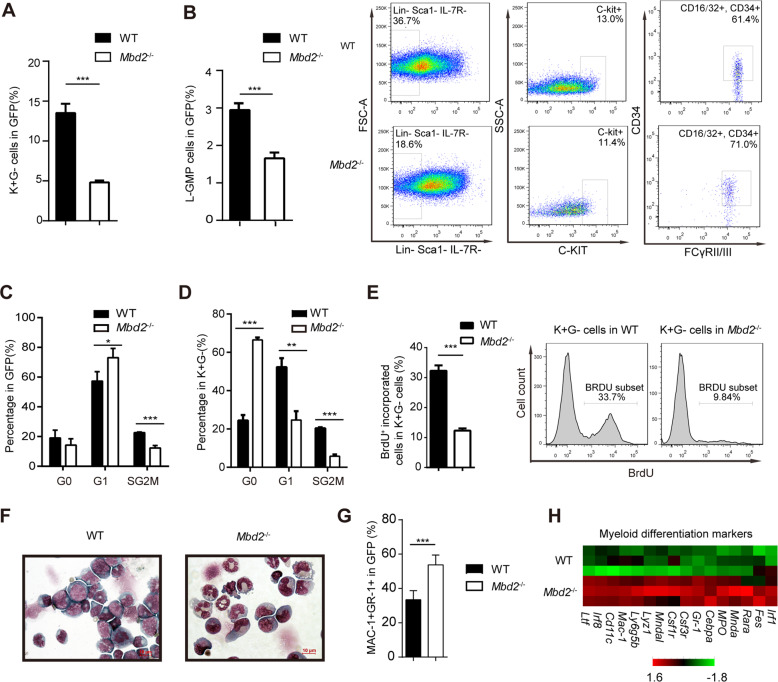
MBD2 maintains the self-renewal property and inhibits the differentiation of LSCs. **A** Flow cytometric determination of the percentage of c-Kit^+^Gr-1^−^ (K^+^G^−^) cells in the BM of leukemic mice (*n* = 5 per group). Error bars, s.d. **B** Left, Flow cytometric determination of the percentage of L-GMP cells in the BM of leukemic mice (*n* = 5 per group). Right, representative FACS plots of L-GMP in the GFP^+^ population. Error bars, s.d. **C**, **D** The cell cycle profile of WT or *Mbd2*^−^^/−^ AML GFP^+^ cells (**C**) and K^+^G^−^ cells (**D**) was determined by flow cytometry. Percentages ± s.d. of cells in different cell cycle phases are shown. **E** The mean percentage ± s.d. values of BrdU^+^ cells are shown within the total K^+^G^−^ leukemia cells (left panel) and representative histograms (right panel) for the cohort (*n* = 5 per group) of *Mbd2*^−/−^ versus WT leukemic mice. **F** Morphology of leukemia cells in BM from either the WT or *Mbd2*^−/−^ group. Cells were stained with Wright–Giemsa. Scale bars represent 10 µm. **G** Flow cytometric determination of the percentage of Mac-1^+^Gr-1^+^ cells (relatively mature/differentiated myeloid cells) among *Mbd2*^−/−^ leukemia cells and WT cells. Error bars, s.d. **H** The expression levels of myeloid differentiation-associated transcripts were determined by qRT-PCR in the indicated cells. *Gapdh* was included as a loading control. *P < 0.05, **P < 0.005, and ***P < 0.001.

The LSC frequency is thought to be associated with prognosis in AML patients and leukemia progression in mice. To identify the potential mechanisms by which MBD2 regulates the stemness of LSCs, we further analyzed the frequency of LSCs from *Mbd2*^*−*/−^ and WT recipients. Previous studies applying MLL-AF9 AML models confirmed that LSCs are enriched in c-Kit^+^Gr-1^−^ (K^+^G^−^) or leukemic granulocyte–macrophage progenitor (L-GMP) populations. We found that loss of MBD2 reduced the proportions of the K^+^G^−^ or L-GMP populations, in accordance with the reduced colony-formatting ability of *Mbd2*^−/−^ LSCs, indicating that MBD2 deletion led to a notable decrease in LSC expansion (Fig. 3A, B and Supplementary Fig. S3G). Consistently, serial in vivo transplantation (P1, P2, and P3 generations) showed a progressive delay of leukemogenesis in the *Mbd2*^−/−^ mice when compared with WT mice (Supplementary Fig. S3A, B and Fig. 2A). The survival of *Mbd2*^−/−^ leukemic mice was remarkably prolonged via serial transplantation compared with the WT groups. Further, the frequency of LSCs, as determined by flow cytometry and serial transplantation, decreased significantly in *Mbd2*^−/−^ mice when compared with WT mice [17].

To determine how MBD2 regulates the proliferation of LSCs, we assessed the cell cycle status of leukemia cells. Cell cycle analysis showed a remarkable decrease in the proportion of actively cycling cells and a notable increase in the proportion of G0/G1-phase cells for both leukemia cells and the LSC-enriched population (Fig. 3C, D). The frequency of actively proliferating LSCs was also reduced after the loss of MBD2 according to the BrdU incorporation studies in vivo (Fig. 3E). MBD2 depletion led to G0/G1 arrest, indicating that the self-renewal capacity of LSCs was weakened by MBD2 deletion (Supplementary Fig. S3A, B, G and Fig. 2A). These data suggested that MBD2 may control cell cycle entry to sustain the LSC pool.

Furthermore, in comparison with their WT counterparts, GFP^+^ leukemia cells of *Mbd2*^−/−^ mice presented more leukemic blast cells with segmented nuclei in the morphological analysis, which suggested increased myeloid differentiation after MBD2 deletion (Fig. 3F). Flow cytometric analysis also demonstrated that MBD2 depletion increased the expression of myeloid differentiation markers Mac-1 and Gr-1 in the *Mbd2*^−/−^ group since Mac-1^+^/Gr-1^+^ cells represent a more mature leukemia cell population (Fig. 3G). The mRNA expression of myeloid differentiation-related genes such as *Mac-1*, *Mpo*, *Gr-1*, etc. was also upregulated after MBD2 deletion (Fig. 3H). Therefore, our data demonstrate that MBD2 depletion enhances myeloid differentiation of leukemia cells and probably leads to delayed AML progression.

### MBD2 ablation leads to a reversal of MLL leukemia-associated gene signatures associated with myeloid differentiation

To investigate the mechanism underlying the longer latency of leukemogenesis caused by the loss of MBD2, leukemic cells were obtained from mice transplanted with WT or *Mbd2*^−/−^ AML cells for microarray analysis to compare their global gene expression profiles. A total of 1878 candidate differentially expressed genes (DEGs) were characterized by comparing the *Mbd2*−/− and WT groups according to the cut-offs *P* value < 0.01 and fold change >1.5 (Supplementary Fig. S4A). As shown in Supplementary Fig. S4B, the candidate genes were significantly enriched for cell cycle genes, LSC signatures, and myeloid differentiation signatures according to Gene Ontology (GO) analysis with the Database for Annotation, Visualization, and Integrated Discovery (DAVID) gene annotation tool.

**Fig. 4:**
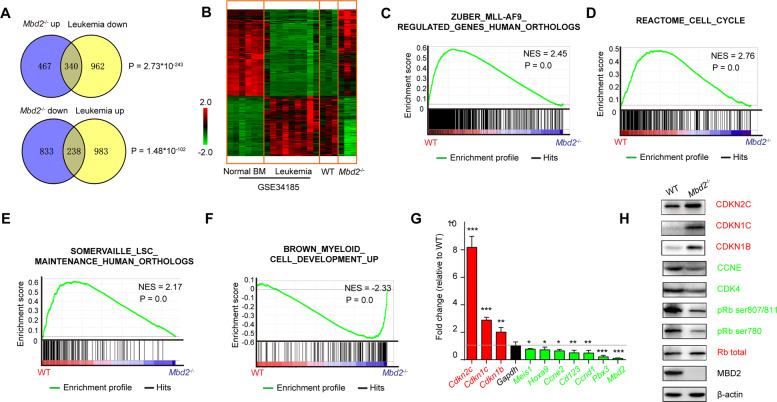
MBD2 ablation partially reverses the MLL leukemia-associated gene signature and promotes myeloid differentiation, possibly by relieving the transcriptional suppression of cell cycle inhibitors. **A** Venn diagrams displaying the intersection of reverse DEGs in WT and *Mbd2*^−/−^ AML cells from our microarray (blue circle, “*Mbd2*^−/−^ up” and “*Mbd2*^−/−^ down” represent the upregulated or downregulated genes in *Mbd2*^−/−^ leukemic cells, respectively), and DEGs in leukemia and normal BM from the GSE34185 dataset (yellow circle, “leukemia up” and “leukemia down” represent the upregulated or downregulated genes in AML cells, respectively). **B** Unsupervised hierarchical clustering was performed using the common DEGs from the GSE34185 dataset and our microarray dataset. **C**–**F** GSEA shows decreased expression of the MLL-AF9 leukemia signature (**C**), downregulation of the cell cycle signature (**D**), loss of the leukemia self-renewal gene expression program (**E**), and increased expression of the myeloid differentiation signature (**F**) in *Mbd2*^−/−^ cells relative to WT cells. NES, normalized enrichment score. **G** Validation of the microarray data of typical cell cycle regulators and MLL rearrangement target genes by qRT-PCR. The level of transcripts in WT AML cells was set at 1.0. Bars represent the mean ± s.d. of two independent experiments. **H** Expression levels of typical cell cycle regulators in WT or *Mbd2*^−/−^ MLL-AF9 cells were determined by WB. β-Actin was used as an internal control. *P < 0.05, **P < 0.005, and ***P < 0.001.

To gain further insight into the role of MBD2 in mediating the MLL-AF9 oncogenic program, we performed a third, unrelated statistical analysis by combining our dataset with GSE34185 dataset [18], which was assessed to determine the DEGs between WT MLL-AF9 leukemic cells and normal mouse bone marrow (BM) cells (Fig. 4A and Supplementary Fig. S4C). With Venny 2.0.2, we identified 660 genes that were differentially expressed in both data sets, with the majority (578 genes, called “reverse DEGs” below) showing a reversal of leukemia-associated expression changes in the *Mbd2*^−/−^ cells (*P* = 1×10^−16^, calculated using the R package SAGx_1.32.0 under R version 2.15.3). The probe sets of reverse DEGs were used to perform unsupervised two-dimensional hierarchical clustering on these merged datasets. WT leukemia samples from our study clustered with leukemia samples from the GSE34185 dataset, whereas *Mbd2*^−/−^ samples from our study clustered with normal BM samples from the GSE34185 dataset (Fig. 4B). Our microarray analysis indicated that MBD2 ablation partially reverses the expression of MLL leukemia-associated gene signature.

Next, to further explore the global genetic changes caused by MBD2 deletion, we conducted gene-set enrichment analysis (GSEA). Consistent with the DAVID analysis results, the GSEA plot showed downregulation of the cell cycle signature, decreased expression of an LSC signature, and increased expression of the myeloid differentiation signature in *Mbd2*^−/−^ leukemia cells compared with control cells (Fig. 4C–F and Supplementary Fig. S4D, E). In addition, the MBD2-related gene signature was associated with better survival in AML patients (Supplementary Fig. S4F).

Together, the data strongly suggest that MBD2 plays an important role in maintaining stem cell-related properties in LSCs, and *Mbd2*^−/−^ leukemic cells show a higher tendency than WT cells to activate the transcriptional program such as those employed during the maturation of myeloid cells.

### Loss of MBD2 attenuates the growth of mouse and human AML cells, possibly by suppressing the cell cycle

Next, the microarray results were validated by real-time reverse transcription-PCR (qRT-PCR), which verified the upregulation of cyclin-dependent kinase inhibitor (CDKI) genes (*Cdkn1b*, *Cdkn1c*, and *Cdkn2c*) and downregulation of self-renewal genes in LSCs (*Meis1*, *Hoxa9*, *Ccne2*, *Cd123, Ccnd1*, and *Pbx3*) after MBD2 deletion (Fig. 4G). Western blotting (WB) was performed using antibodies against the cell cycle and epigenetic regulators. We found that MBD2 deletion could upregulate the CDKI locus (CDKN1B, CDKN1C, and CDKN2C), downregulate CCNE and CDK4 activity, decrease the phosphorylation of pRb at Ser780 (a site targeted by cyclin D-CDK4), decrease the phosphorylation of pRb at Ser807/811 (a site targeted by cyclin E-CDK2), and increase the levels of total pRb (Fig. 4H and Supplementary Fig. S5A). In addition, MBD2 deletion did not affect H3K79me2 or other histone methylation sites (Supplementary Fig. S5B).

**Fig. 5:**
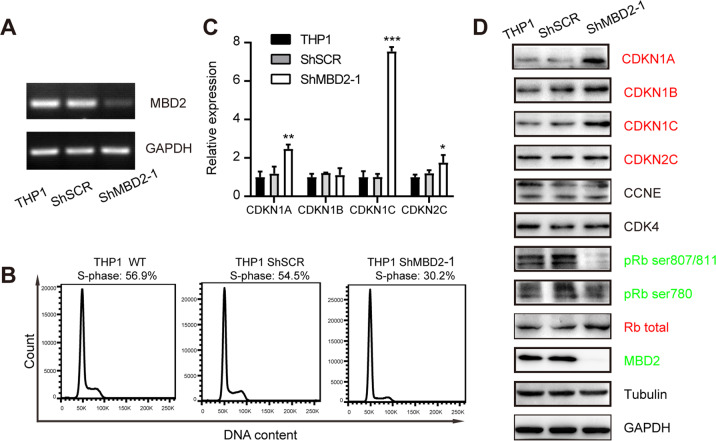
Downregulation of MBD2 attenuates leukemogenesis in THP1 human AML cells by activating cell cycle inhibitors. **A** The expression of *MBD2* transcripts was determined by qRT-PCR in parental THP1 and THP1 shSCR and shMBD2-1 cells. *GAPDH* was included as a loading control. **B** Typical flow cytometric profiles show representative cell cycle data from two independent experiments. The cell cycle was analyzed using PI staining. **C** qRT-PCR indicated the transcript level of cell cycle regulators in the depicted cells. Error bars, s.d. **D** Expression levels of typical cell cycle regulators in parental THP1, THP1 shSCR, and shMBD2-1 cells were determined by WB. GAPDH was used as an internal control. *P < 0.05, **P < 0.005, and ***P < 0.001.

We next assessed the functional role of MBD2 in human MLL-rearranged leukemia. THP1 shMBD2 cells were verified to have reduced mRNA expression of MBD2 compared with that in THP1 shSCR cells (Fig. 5A). Flow cytometry data showed that 56.9% of parental THP1 cells, 54.5% of THP1 shSCR cells, and 30.2% of THP1 shMBD2-1 cells were in the S phase of the cell cycle, suggesting that the cell cycle was arrested at the G0/G1 phase after MBD2 depletion (Fig. 5B). qRT-PCR suggested that the transcription levels of the cell cycle inhibitors *CDKN1B*, *CDKN1C*, and *CDKN2C* were higher after MBD2 knockdown (Fig. 5C). WB also showed significantly elevated levels of CDKN1A, CDKN1B, CDKN1C, CDKN2C, and total Rb protein and decreased phosphorylation levels of pRb at Ser780 and pRb at Ser807/811 in THP1 shMBD2-1 cells compared with control cells (Fig. 5D). Cell cycle and WB analysis of THP-1 cells after MBD2 knockdown by a second shRNA, shMBD2-2 displayed similar results with that of THP-1 shMBD2-1 (Supplementary Fig. S6A, B). These data mirrored those from genetic mouse experiments, and this model revealed that MBD2 exerts an antiproliferative effect via cell cycle arrest by increasing CDKI expression.

### MBD2 suppresses the transcriptional expression of CDKN1C probably by binding to its promoter in leukemic cells

**Fig. 6:**
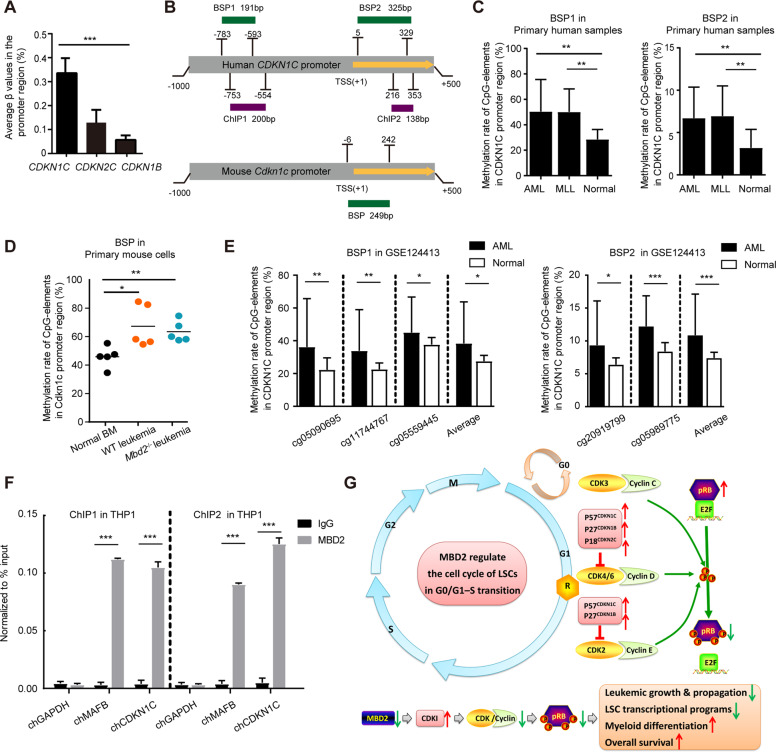
MBD2 may suppress the transcriptional expression of CDKN1C by binding to its promoter in leukemic cells. **A** Average β values of *CDKN1B* (P27), *CDKN1C* (P57), and *CDKN2C* (P18) in AML patients from the TCGA dataset. Error bars, s.d. **B** Schematic representation of the *CDKN1C* promoter in humans and mouse, respectively. Short horizontal lines represent corresponding positions amplified by bisulphite sequencing PCR (BSP) and chromatin immunoprecipitation (ChIP) primers. Upper panel, the human *CDKN1C* promoter region. Lower panel, the mouse *Cdkn1c* promoter region. TSS, transcription start site as +1 position. **C** The *CDKN1C* methylation levels for the location of the CpG islands (BSP1 and BSP2 regions) in normal human BM cells (*n* = 11), AML patients (*n* = 19), and MLL patients (*n* = 9) are shown. Error bars, s.d. BSP1 position, 191 bp, 2,907,589–2,907,779 (hg19); BSP2 position, 325 bp, 2,906,667–2,906,991 (hg19). **D** The average *Cdkn1c* methylation levels of WT and *Mbd2*^−/−^ murine leukemic cells from the P3 generation. Mouse BSP position, 249 bp, 143,460,819–143,461,067 (mm10). **E** Methylation analysis of the five CG sites (BSP1: left panel, cg05090695, cg05559445, cg11744767; BSP2: right panel, cg05989775, cg20919799) in AML patients (*n* = 459) and healthy control cases (*n* = 41) from public GEO database GSE124413. The results are expressed as the mean ± s.d. **F** Binding of MBD2 to the *CDKN1C* promoter was examined by chromatin immunoprecipitation assay (ChIP) in THP1 cells. MAFB was selected as a positive control, and GAPDH was selected as a negative control. Bars represent the mean ± s.d. percentages for two replicates per ChIP assay. ChIP1 position, 200 bp, 2,907,550–2,907,749 (hg19); ChIP2 position, 138 bp, 2,906,643–2,906,780 (hg19). **G** Model depicting the role of MBD2 in AML and its effect on the cell cycle of leukemia cells. Red arrows, increased expression after MBD2 deletion; green arrows, decreased expression after MBD2 deletion. *P < 0.05, **P < 0.005, and ***P < 0.001.

Previous studies have shown that MBD2 functions as a “reader” of DNA methylation and translates methylated DNA into signals for transcriptional silencing [9, 11, 19, 20]. Therefore, we reasoned that the transcript levels of CDKIs might be consequently restored after the loss of MBD2. Indeed, CDKN1C had a higher methylation level than CDKN1B and CDKN2C according to the analysis of AML patients from The Cancer Genome Atlas (TCGA) database (Fig. 6A). We assessed the methylation levels of CDKN1C using bisulfite sequencing PCR (BSP) in human AML samples and murine cells (Fig. 6B). MethPrimer 2.0 and Integrative Genomics Viewer showed the precise positions of BSP1 and BSP2 in the promoter region of CDKN1C in humans and mice (Supplementary Fig. S7A–D), respectively. Compared with the corresponding healthy controls, significantly higher methylation levels of CDKN1C in the same region were detected in AML cells from both primary human and mouse samples (Fig. 6C, D). To independently validate the findings obtained in our study, AML patients (*n* = 459) and healthy control cases (*n* = 41) with matched age or sex from Gene Expression Omnibus (GEO) database GSE124413 were used [21]. The methylation levels of five CG sites in CDKN1C gene promoter are significantly higher in AML patients than in the control (Fig. 6E). Interestingly, the methylation status of these promoter-associated CpG-rich regions remained remarkably stable after the loss of MBD2 in murine and human AML cells (Fig. 6D and Supplementary Fig. S8A).

Next, to identify the specific position where MBD2 binds to *CDKN1C* promoter, we constructed the corresponding chromatin immunoprecipitation (ChIP) assay primers for differentially methylated regions of *CDKN1C* promoter based on the results of BSP sequencing (Fig. 6B and Supplementary Fig. S7C). The ChIP results supported that the methylation of the *CDKN1C* promoter region might be read by MBD2 in AML; MAFB was used as a positive control and GAPDH was used as a negative control (Fig. 6F). The transcriptional silencing of the abnormally methylated *CDKN1C* promoter region could be effectively relieved by knockdown of MBD2 (Fig. 5C, D and Supplementary Fig. S6B). In line with these findings, LSCs from the human AML dataset GSE24006 also correlated with high MBD2 expression and low *CDKN1C* expression [22] (Supplementary Fig. S8B). Furthermore, analysis with the cBioPortal online tool revealed that though the average methylation levels of CDKN1C in AML patients with a poor prognosis had no significant differences versus those patients with a favorable prognosis from the TCGA database, low expression levels of CDKN1C were unfavorable prognostic factors in AML, indicating the potential of CDKN1C as a prognostic indicator (Supplementary Fig. S8C–E). Therefore, the transcriptional expression of CDKN1C was probably suppressed by MBD2 through binding to its promoter in leukemic cells.

Taken together, these data indicate that the observed proliferative arrest of *Mbd2*^−/−^ AML cells resulted from cell cycle blockade and that the impaired self-renewal capacity of LSCs was associated with increased *CDKN1C* expression (Fig. 6G).

## Discussion

Clinically eradicative treatment in AML patients still has limited success, probably due to the existence of LSCs. Previous data have suggested that MBD2 silences key tumor suppressors that might influence tumoral stemness in several cancers [9–11, 19, 20]. In this study, we demonstrated that MBD2 deletion delayed MLL-AF9 leukemia onset. Serial transplantation experiments showed that MBD2 depletion impaired the self-renewal capacity of LSCs and promoted myeloid differentiation. Mechanistically, we propose that the methylation reader MBD2 constitutes a novel upstream mechanism that suppresses *CDKN1C* expression and regulates LSC oncogenic potential in MLL-rearranged AML.

Many studies have demonstrated that de-repression of negative regulators for stem cell maintenance could lead to a loss of the self-renewal capacity of LSCs over time. We reasoned that MBD2 promotes LSC cell cycle progression probably by inhibiting negative regulators of transcription. The obvious inverse relationship between cell cycle activity and self-renewal capacity in stem cells makes CDKIs the core of candidate genes that may maintain self-renewal [23]. Among CDKIs, CDKN1C has been shown to play a dominant role in the maintenance of stem cells [24]. In our study, cell cycle analysis demonstrated that compared to the control conditions, MBD2 deletion led to an increase in the proportion of G0/G1-phase cells. We observed proliferative arrest in *Mbd2*^−/−^ cells due to a cell cycle blockade associated with increased expression of CDKN1C in our human and mouse experiments. DNA methylation participates in CDKN1C silencing during tumorigenesis, especially in hematological malignancies such as ALL [25], large B cell lymphoma [26], and AML [27]. Our BSP analysis demonstrated that the methylation levels of *CDKN1C* promoter are significantly higher in AML patients than that of the healthy controls, and MBD2 deficiency did not affect the methylation status of *CDKN1C* promoter regions in leukemia cells.

Using the corresponding ChIP primers of *CDKN1C* promoter for differentially methylated regions based on the BSP sequencing result for ChIP assay, we showed that MBD2 acts as a DNA methylome reader by binding with the methylated promoter-associated CpG islands of *CDKN1C* as previously reported in HeLa cells [28]. MBD2 expression was negatively correlated with *CDKN1C* expression in clinical myeloid leukemia samples [22]. Moreover, treatment of tumor cell lines with demethylating agents such as 5-aza-2′-deoxycytidine resulted in *CDKN1C* expression activation [29]. Thus, the epigenetic changes in CDKN1C *cis*-regulatory elements caused by epigenetic drugs could potentially be a way for therapeutic intervention of CDKN1C expression. Although the DNA methylation levels of different CG sites in the *CDKN1C* promoter are distinct (BSP1 and BSP2 positions), significantly increased methylation of *CDKN1C* in the same region was detected in AML cells, when compared with healthy control cells. This might be because the *CDKN1C* gene is almost entirely contained in a CpG island that extends from ~600 bp upstream of the transcriptional start site and into the gene body [30, 31], and the restricted sequences analyzed by BSP made it difficult to evaluate the total methylation level of *CDKN1C*.

The induction of differentiation in AML stem cells has already been confirmed to be an effective method for leukemia treatment. Cellular differentiation commonly involves a tight coupling of withdrawal from the cell cycle [32]. The increased accumulation of cells in the G0/G1 phase might make the cells more sensitive to other differentiation stimuli. In our study, loss of MBD2 exerted an antiproliferative effect via cell cycle arrest, significantly induced surface antigens related to myeloid cell maturation, and led to morphological changes indicative of differentiation. These phenotypic changes were paralleled by increased expression of *Gr-1*, *Ltf*, *Irf8*, etc. [16, 33, 34], which encode transcription factors involved in myelomonocytic differentiation, and increased accessibility of the self-renewal genes *Pbx3*, *Cd123*, *Meis1*, etc. [35–37] in LSCs. Moreover, both gene expression analyses and multidimensional signaling studies showed that MBD2 negatively modulated the genes related to myeloid differentiation and was necessary to maintain an expression program related to MLL-induced leukemogenesis. Together, we speculate that lack of MBD2 effectively causes the collapse of stemness genetic program, reduces the number of functionally defined LSCs, reverses the developmental arrest induced by oncogenes, and thereby allows LSCs to enter a more mature state, reflecting normal BM production.

In summary, our data revealed that MBD2 deletion could delay the initiation and development of MLL-AF9 leukemia and promote LSC differentiation. MBD2 might modulate *CDKN1C* expression by binding to its promoter regions in leukemic cells. Our results may also apply to distinct AML subsets since no significant difference was observed in the MBD2 expression pattern among different cytogenetic/molecular AML subgroups according to the analysis of a web-based and repository of data from the Oncomine and Bloodspot databases. Our data suggest that further development of specific inhibitors for MBD2 is warranted to evaluate the potential of MBD2 as a new therapeutic target in drug combination therapies for various subtypes of myeloid malignancies.

## Materials and methods

### Mice and genotyping

MBD2-deficient (*Mbd2*^−/−^) mice on a C57BL/6 background were obtained from Dr. Adrian Bird (Edinburgh University, Edinburgh, UK) [38]. All animal studies performed were approved by the Institutional Committee of Animal Care and Treatment of Tongji Hospital. All mice were reared in a pathogen-free animal facility of Tongji Hospital affiliated with Huazhong University of Science and Technology, China. This study used 6- to 8-week-old male *Mbd2*^−/−^ mice and WT littermates. Genotyping was performed as described previously [12, 39].

### Murine MLL-AF9 AML model and serial transplantation experiments

MSCV-MLL-AF9-GFP plasmids were used to produce MLL-AF9 retroviruses by a method reported previously [40]. C-kit^+^ cells obtained from WT or *Mbd2*^−/−^ mice were infected with MLL-AF9 retrovirus and immortalized in in vitro culture. GFP^+^ cells were isolated after 72 h of infection and injected intravenously into lethally irradiated recipient mice (P0). Serial transplantation was performed to compare the self-renewal ability of the established leukemia cells from two groups. We defined the GFP^+^ cells of primary AML mice as P0 cells and intravenously injected them into P1 sublethally irradiated recipients. Leukemic cells were subsequently expanded to generate the P2 generation and then to the P3 generation.

### Cell lines and clinical specimens

THP1 cells (bearing the MLL-AF9 fusion protein) were originally purchased from the American Type Culture Collection (Manassas, USA), verified by short tandem repeat profiling and directly cultured for no more than 3 months to ensure that they did not contain mycoplasma.

For AML samples, the Ficoll gradient method was used to separate WBCs from BM specimens obtained before chemotherapy, and the obtained cells were cryopreserved. In accordance with the principles expressed in the Declaration of Helsinki, a written informed consent form was obtained from each patient and healthy volunteer, and the study was approved by the Institutional Review Committee of the Use of Human Materials in Tongji Hospital.

### Microarray and bioinformatics analysis

A fluorescence-activated cell sorter (Aria Cell Sorter, BD Biosciences) was used to sort the specific cell population. The Affymetrix GeneChip Mouse Gene 2.0 ST Array was used to explore the expression changes in the entire transcriptome. DEGs were statistically defined by two-group *t* test; genes were selected based on an average fold change >1.5 and a *P* value < 0.01 for the comparison of WT and *Mbd2*^−/−^ leukemia cells. Microarray raw data were uploaded to the GEO repository as GSE166610. Next, the microarray results were validated by qRT-PCR. The sequences of all primers are listed in Supplementary Table S1.

To further explore the role of MBD2 in mediating leukemogenesis, we compared the DEGs from our microarray data with the MLL-AF9-associated gene signatures from the GEO database. The MLL-AF9-associated gene signatures were obtained from GSE34185 [18], which compared the gene profiles of WT MLL-AF9 AML cells with normal BM cells. Then, we used the Venn diagram network tool to draw the Venn diagram (https://bioinfogp.cnb.csic.es/tools/venny/). The distinct microarray data profiles are represented as different color areas. The cross areas indicate the overlapping DEGs. The *P* value was calculated by exact hypergeometric probability. Next, each individual dataset was transformed independently relative to its own average value, and then the two datasets were combined [41]. Based on Cluster 3.02 software, overlapping DEGs were used to perform unsupervised two-dimensional hierarchical clustering on these merged datasets.

The DAVID (https://david.ncifcrf.gov/) [42] was used to perform GO enrichment analysis for the upregulated and downregulated genes. To identify the classes of genes significantly regulated by MBD2, GSEA of the Molecular Signatures Database (https://www.broadinstitute.org/msigdb) [43] combined with several relevant gene sets (published tumor self-renewal signatures or myeloid differentiation signatures) was performed.

### MBD2 knockdown by customized CRISPR/Cas9 vector

Since small interfering RNA knockdown failed to reduce MBD2 protein expression more than 50% in THP1 cells, lentiviruses including the Cas9 system and MBD2 single guide RNAs (sgRNAs) (shMBD2) or scramble sgRNA (shSCR) were used to transfect THP1 cells as previously reported [12, 39]. Oligo-DNA targeting the MBD2 exon 2 locus was designed by the Zhang Feng Laboratory of Massachusetts Institute of Technology (MIT) online software http://crispr.mit.edu/. GeneChem (Shanghai, China) cloned the relevant sgRNAs into the GV393 plasmid (U6-sgRNA-EF1a-Cas9-FLAG-P2A-EGFP). The sequence of shMBD2-1 was CCTCAGTTGGCAAGGTACCT, the sequence of shMBD2-2 was GGCAAGAGCGATGTCTACTACTTCATTCAAGAGA, and the sequence of shSCR was CGCTTCCGCGGCCCGTTCAA.

### BSP analysis and ChIP assay

BSP sequencing was performed as previously reported [12, 39]. After obtaining the transformed DNA, the *CDKN1C* promoter region was amplified. The mouse primers used were as follows: forward, 5′-GGTGTTGTTGAAATTGAAAA-3′ and reverse, 5′-ATAAAACCCCTTACACAACC-3′. The human primers used were as follows: BSP1, forward 5′-GGTTTTGGTTTGYGTYGGATGGGGTT-3′ and reverse, 5′-TCAAAATATCCCACACATAATCCCTC-3′; BSP2, forward 5′-GTTGGGYGTTTTATAGGTTAAG-3′ and reverse, 5′-CACTAATACTAAAAAAATCCCAC-3′.

For ChIP assays, the purified DNA was removed to amplify the DNA sequences targeted by MBD2 using a ChIP Assay Kit (Millipore) as described previously [12, 39]. The primers used in ChIP assays are summarized in Supplementary Table S2.

MethPrimer 2.0 [44] (http://www.urogene.org/methprimer2/index.html) and Integrative Genomics Viewer [45] (http://software.broadinstitute.org/software/igv/) were used to illustrate the schematic diagram of the CpG island and the precise position of BSP and ChIP in the promoter region of CDKN1C in humans and mice, respectively.

### Statistical analysis

Statistical analysis was performed using GraphPad Prism 6.0 and SPSS 21.0 software. For bar graphs, unpaired two-tailed Student’s *t* tests were constructed to calculate *P* values. Kaplan–Meier survival curves were obtained and log-rank statistical analysis was performed to calculate *P* values. *P* < 0.05 was considered to indicate significance.

Additional experimental procedures are provided in the Supplemental Materials and methods.

## Supplementary information

Supplemental Information

## References

[1] Short NJ, Rytting ME, Cortes JE (2018). Acute myeloid leukaemia. Lancet.

[2] Thomas D, Majeti R (2017). Biology and relevance of human acute myeloid leukemia stem cells. Blood.

[3] Eppert K, Takenaka K, Lechman ER, Waldron L, Nilsson B, van Galen P (2011). Stem cell gene expression programs influence clinical outcome in human leukemia. Nat Med.

[4] Sykes DB, Kfoury YS, Mercier FE, Wawer MJ, Law JM, Haynes MK (2016). Inhibition of dihydroorotate dehydrogenase overcomes differentiation blockade in acute myeloid leukemia. Cell.

[5] Pan D, Rampal R, Mascarenhas J (2020). Clinical developments in epigenetic-directed therapies in acute myeloid leukemia. Blood Adv.

[6] Cai SF, Levine RL (2019). Genetic and epigenetic determinants of AML pathogenesis. Semin Hematol.

[7] Mahmood N, Rabbani SA (2019). DNA methylation readers and cancer: mechanistic and therapeutic applications. Front Oncol.

[8] Sansom OJ, Maddison K, Clarke AR (2007). Mechanisms of disease: methyl-binding domain proteins as potential therapeutic targets in cancer. Nat Clin Pract Oncol.

[9] Gong W, Ni M, Chen Z, Zheng Z (2020). Expression and clinical significance of methyl-CpG binding domain protein 2 in high-grade serous ovarian cancer. Oncol Lett.

[10] Li L, Li N, Liu N, Huo F, Zheng J (2020). MBD2 correlates with a poor prognosis and tumor progression in renal cell carcinoma. OncoTargets Ther.

[11] Stefanska B, Suderman M, Machnes Z, Bhattacharyya B, Hallett M, Szyf M (2013). Transcription onset of genes critical in liver carcinogenesis is epigenetically regulated by methylated DNA-binding protein MBD2. Carcinogenesis.

[12] Zhou M, Zhou K, Cheng L, Chen X, Wang J, Wang XM (2018). MBD2 ablation impairs lymphopoiesis and impedes progression and maintenance of T-ALL. Cancer Res.

[13] Milne TA (2017). Mouse models of MLL leukemia: recapitulating the human disease. Blood.

[14] Metzeler KH, Herold T, Rothenberg-Thurley M, Amler S, Sauerland MC, Görlich D (2016). Spectrum and prognostic relevance of driver gene mutations in acute myeloid leukemia. Blood.

[15] Johnson JJ, Chen W, Hudson W, Yao Q, Taylor M, Rabbitts TH (2003). Prenatal and postnatal myeloid cells demonstrate stepwise progression in the pathogenesis of MLL fusion gene leukemia. Blood.

[16] Kumar AR, Hudson WA, Chen W, Nishiuchi R, Yao Q, Kersey JH (2004). Hoxa9 influences the phenotype but not the incidence of Mll-AF9 fusion gene leukemia. Blood.

[17] Park SM, Cho H, Thornton AM, Barlowe TS, Chou T, Chhangawala S (2019). IKZF2 drives leukemia stem cell self-renewal and inhibits myeloid differentiation. Cell Stem Cell.

[18] Li Z, Huang H, Chen P, He M, Li Y, Arnovitz S (2012). miR-196b directly targets both HOXA9/MEIS1 oncogenes and FAS tumour suppressor in MLL-rearranged leukaemia. Nat Commun.

[19] Martin V, Jorgensen HF, Chaubert AS, Berger J, Barr H, Shaw P (2008). MBD2-mediated transcriptional repression of the p14ARF tumor suppressor gene in human colon cancer cells. Pathobiology.

[20] Mian OY, Wang SZ, Zhu SZ, Gnanapragasam MN, Graham L, Bear HD (2011). Methyl-binding domain protein 2-dependent proliferation and survival of breast cancer cells. Mol Cancer Res.

[21] Bolouri H, Farrar JE, Triche T, Ries RE, Lim EL, Alonzo TA (2018). The molecular landscape of pediatric acute myeloid leukemia reveals recurrent structural alterations and age-specific mutational interactions. Nat Med.

[22] Gentles AJ, Plevritis SK, Majeti R, Alizadeh AA (2010). Association of a leukemic stem cell gene expression signature with clinical outcomes in acute myeloid leukemia. JAMA.

[23] Hao S, Chen C, Cheng T (2016). Cell cycle regulation of hematopoietic stem or progenitor cells. Int J Hematol.

[24] Tesio M, Trumpp A (2011). Breaking the cell cycle of HSCs by p57 and friends. Cell Stem Cell.

[25] Shen L, Toyota M, Kondo Y, Obata T, Daniel S, Pierce S (2003). Aberrant DNA methylation of p57KIP2 identifies a cell-cycle regulatory pathway with prognostic impact in adult acute lymphocytic leukemia. Blood.

[26] Li Y, Nagai H, Ohno T, Yuge M, Hatano S, Ito E (2002). Aberrant DNA methylation of p57(KIP2) gene in the promoter region in lymphoid malignancies of B-cell phenotype. Blood.

[27] Brakensiek K, Länger F, Kreipe H, Lehmann U (2005). Absence of p21(CIP 1), p27(KIP 1) and p 57(KIP 2) methylation in MDS and AML. Leuk Res.

[28] Chatagnon A, Perriaud L, Nazaret N, Croze S, Benhattar J, Lachuer J (2011). Preferential binding of the methyl-CpG binding domain protein 2 at methylated transcriptional start site regions. Epigenetics.

[29] Sanaei M, Kavoosi F (2019). Effect of 5-aza-2’-deoxycytidine in comparison to valproic acid and trichostatin A on histone deacetylase 1, DNA methyltransferase 1, and CIP/KIP family (p21, p27, and p57) genes expression, cell growth inhibition, and apoptosis induction in colon cancer SW480 cell line. Adv Biomed Res.

[30] Rossi MN, Andresini O, Matteini F, Maione R (2018). Transcriptional regulation of p57(kip2) expression during development, differentiation and disease. Front Biosci.

[31] Stampone E, Caldarelli I, Zullo A, Bencivenga D, Mancini FP, Della Ragione F (2018). Genetic and epigenetic control of CDKN1C expression: importance in cell commitment and differentiation, tissue homeostasis and human diseases. Int J Mol Sci.

[32] Blomen VA, Boonstra J (2007). Cell fate determination during G1 phase progression. Cell Mol Life Sci.

[33] Li Z, Luo RT, Mi S, Sun M, Chen P, Bao J (2009). Consistent deregulation of gene expression between human and murine MLL rearrangement leukemias. Cancer Res.

[34] Marschalek R (2011). Mechanisms of leukemogenesis by MLL fusion proteins. Br J Haematol.

[35] Liu K, Zhu M, Huang Y, Wei S, Xie J, Xiao Y (2015). CD123 and its potential clinical application in leukemias. Life Sci.

[36] Wong P, Iwasaki M, Somervaille TC, So CW, Cleary ML (2007). Meis1 is an essential and rate-limiting regulator of MLL leukemia stem cell potential. Genes Dev.

[37] Guo H, Chu Y, Wang L, Chen X, Chen Y, Cheng H (2017). PBX3 is essential for leukemia stem cell maintenance in MLL-rearranged leukemia. Int J Cancer.

[38] Hendrich B, Guy J, Ramsahoye B, Wilson VA, Bird A (2001). Closely related proteins MBD2 and MBD3 play distinctive but interacting roles in mouse development. Genes Dev.

[39] Cheng L, Tang Y, Chen X, Zhao L, Liu S, Ma Y (2018). Deletion of MBD2 inhibits proliferation of chronic myeloid leukaemia blast phase cells. Cancer Biol Ther.

[40] Zheng Y, Zhang H, Wang Y, Li X, Lu P, Dong F (2016). Loss of Dnmt3b accelerates MLL-AF9 leukemia progression. Leukemia.

[41] Sachs Z, LaRue RS, Nguyen HT, Sachs K, Noble KE, Mohd Hassan NA (2014). NRASG12V oncogene facilitates self-renewal in a murine model of acute myelogenous leukemia. Blood.

[42] Huang DW, Sherman BT, Tan Q, Kir J, Liu D, Bryant D (2007). DAVID Bioinformatics Resources: expanded annotation database and novel algorithms to better extract biology from large gene lists. Nucleic Acids Res.

[43] Subramanian A, Tamayo P, Mootha VK, Mukherjee S, Ebert BL, Gillette MA (2005). Gene set enrichment analysis: a knowledge-based approach for interpreting genome-wide expression profiles. Proc Natl Acad Sci USA.

[44] Li LC, Dahiya R (2002). MethPrimer: designing primers for methylation PCRs. Bioinformatics.

[45] Thorvaldsdóttir H, Robinson JT, Mesirov JP (2013). Integrative Genomics Viewer (IGV): high-performance genomics data visualization and exploration. Brief Bioinform.

