# Supplementary material for: Loss of MBD2 attenuates MLL-AF9-driven leukemogenesis by suppressing the leukemic cell cycle via CDKN1C
Source: Oncogenesis. 2021 Nov 17;10(11):79. doi: 10.1038/s41389-021-00366-3 (PMC8599466; doi:10.1038/s41389-021-00366-3)
Supplement: Supplementary file 1 — Supplemental Information [file 41389_2021_366_MOESM1_ESM.docx]

**Supplemental Information**

**CONTENTS**

SUPPLEMENTAL MATERIALS AND METHODS

REFERENCES

SUPPLEMENTAL TABLES S1-2

SUPPLEMENTAL FIGURES S1-8

SUPPLEMENTAL LEGENDS OF FIGURES S1-8

**SUPPLEMENTAL MATERIALS AND METHODS**

**The role of MBD2 according to analysis of** **public clinical databases**

To elucidate the role of MBD2 by means of public clinical database analysis, we compared the expression profiles of cancer tissues with normal tissues based on The Cancer Genome Atlas (TCGA) and the Genotype-Tissue Expression Project (GTEx, https://commonfund.nih.gov/GTEx/) data through excavating the Gene Expression Profiling Interactive Analysis (GEPIA) online server (<http://gepia.cancer-pku.cn/>) [1]. Next, we used SurvExpress (<http://bioinformatica.mty.itesm.mx/SurvExpress>) to compare *MBD2* expression in high-risk AML patients versus low-risk AML patients [2]. Oncomine (<https://www.oncomine.org/resource/login.html>) [3] and BloodSpot (<http://servers.binf.ku.dk/bloodspot/>) [4] were used to investigate the expression level of *MBD2* in different FAB classifications and different cytogenetic/molecular subgroups of AML. PrognoScan [5] was used to identify the prognostic role of candidate genes (<http://gibk21.bse.kyutech.ac.jp/PrognoScan/index.html>). Through the cBioPortal for Cancer Genomics (<http://cbioportal.org>) [6] and the University of California, Santa Cruz (UCSC) Xena website (<https://xenabrowser.net/>), we analyzed the DNA methylation level of several candidate genes in the TCGA database.

**Colony formation assay**

Lin^-^, c-Kit^+^, and sca1^+^ bone marrow (BM) cells were isolated from WT or *Mbd2*^-/-^ mice and plated in M3434 methylcellulose (n=3). For leukemia cells or LSCs, isolated GFP^+^ or K^+^G^-^ cells were cultured, and colonies were counted after 7 days of culture by previously reported methods [7]. A previous report indicated that colonies of type I to type II to type III reflected myeloid differentiation maturation, suggesting the progressive loss of stemness in AML cells [8].

**Flow cytometry**

Flow cytometry data were acquired with an LSR II cytometer (BD Biosciences) as described previously [7, 9]. In short, a mixture of antibodies against CD3, B220 (CD45R), Ter119, Mac-1(CD11b), Gr-1 (Ly-6G), CD4, CD8, and IL-7R was used to exclude the lineage mix (Lin). Mac-1 (CD11b) and Gr-1 (Ly-6G) were used for myeloid differentiation. To determine the apoptosis status of leukemia cells, the cells were incubated with antibodies conjugated to surface markers and subsequently stained with 7-aminoactinomycin D (7-AAD) and annexin-V antibodies in binding buffer. Bromodeoxyuridine (BrdU) detection was performed as described previously [10].

**Western blotting (WB) and *q*RT-PCR**

Western blot analysis was performed as reported previously. Commercial antibodies against the following proteins were used: H3K79me2, H3K4me3, H3K27me3, H3K36me3 and H3 (Abcam), MBD2 (Santa Cruz), CDKN1B, CDKN1C, CDKN2C, CCDE, total Rb protein, pRb at Ser780, pRb at Ser807/811, CDKN1A, CDK3, CCNC, CDK6, E2F, CDK2, and β-actin (Cell Signaling Technologies).

Reverse transcription-PCR (*q*RT-PCR) was then conducted according to the protocol of the manufacturer as described previously. The sequences of all primers are listed in Supplementary Table S1.

**REFERENCES**

1 Tang Z, Li C, Kang B, Gao G, Li C, Zhang Z. GEPIA: a web server for cancer and normal gene expression profiling and interactive analyses. Nucleic acids research. 2017; 45: W98-w102.

2 Aguirre-Gamboa R, Gomez-Rueda H, Martínez-Ledesma E, Martínez-Torteya A, Chacolla-Huaringa R, Rodriguez-Barrientos A*, et al*. SurvExpress: an online biomarker validation tool and database for cancer gene expression data using survival analysis. PloS one. 2013; 8: e74250.

3 Rhodes DR, Yu J, Shanker K, Deshpande N, Varambally R, Ghosh D*, et al*. ONCOMINE: a cancer microarray database and integrated data-mining platform. Neoplasia (New York, NY). 2004; 6: 1-6.

4 Bagger FO, Kinalis S, Rapin N. BloodSpot: a database of healthy and malignant haematopoiesis updated with purified and single cell mRNA sequencing profiles. Nucleic acids research. 2019; 47: D881-d85.

5 Mizuno H, Kitada K, Nakai K, Sarai A. PrognoScan: a new database for meta-analysis of the prognostic value of genes. BMC medical genomics. 2009; 2: 18.

6 Gao J, Aksoy BA, Dogrusoz U, Dresdner G, Gross B, Sumer SO*, et al*. Integrative analysis of complex cancer genomics and clinical profiles using the cBioPortal. Science signaling. 2013; 6: pl1.

7 Zheng Y, Zhang H, Wang Y, Li X, Lu P, Dong F*, et al*. Loss of Dnmt3b accelerates MLL-AF9 leukemia progression. Leukemia. 2016; 30: 2373-84.

8 Kumar AR, Hudson WA, Chen W, Nishiuchi R, Yao Q, Kersey JH. Hoxa9 influences the phenotype but not the incidence of Mll-AF9 fusion gene leukemia. Blood. 2004; 103: 1823-8.

9 Cheng H, Hao S, Liu Y, Pang Y, Ma S, Dong F*, et al*. Leukemic marrow infiltration reveals a novel role for Egr3 as a potent inhibitor of normal hematopoietic stem cell proliferation. Blood. 2015; 126: 1302-13.

10 Zhou M, Zhou K, Cheng L, Chen X, Wang J, Wang XM*, et al*. MBD2 Ablation Impairs Lymphopoiesis and Impedes Progression and Maintenance of T-ALL. Cancer Res. 2018; 78: 1632-42.

11 Metzeler KH, Hummel M, Bloomfield CD, Spiekermann K, Braess J, Sauerland MC*, et al*. An 86-probe-set gene-expression signature predicts survival in cytogenetically normal acute myeloid leukemia. Blood. 2008; 112: 4193-201.

**SUPPLEMENTAL TABLES**

**Table S1 - Lists of the sequences for PCR primers used in the studies**

|  | Forward Primer (5'-3') | Reverse Primer (5'-3') |  |  |  |
| --- | --- | --- | --- | --- | --- |
| **Mouse** | | |  |  |  |
| *Irf1* | ATGCCAATCACTCGAATGCG | TTGTATCGGCCTGTGTGAATG |  |  |  |
| *Fes* | CCACCACAACCGCTACGTC | CATCTCCTCGTGCAAATCCTG |  |  |  |
| *Rara* | TTCTTTCCCCCTATGCTGGGT | GGGAGGGCTGGGTACTATCTC |  |  |  |
| *Mnda* | GACAACCAAGAGCAATACACCA | ATCAGTTTGCCCAATCCAGAAT |  |  |  |
| *Cebpd* | CGACTTCAGCGCCTACATTGA | CTAGCGACAGACCCCACAC |  |  |  |
| *Ly6g* | GCTGTCCTCTTAACAGTGCTG | AGGTACTTGTTTAGTGGGAGGG |  |  |  |
| *Csf3r* | CTGATCTTCTTGCTACTCCCCA | GGTGTAGTTCAAGTGAGGCAG |  |  |  |
| *Csf1r* | TGTCATCGAGCCTAGTGGC | CGGGAGATTCAGGGTCCAAG |  |  |  |
| *Lyz1* | GAGACCGAAGCACCGACTATG | CGGTTTTGACATTGTGTTCGC |  |  |  |
| *Ly6g5b* | CGCGTGCTTGTAGGTATGCT | CGAAGGGTCTTCTAAGAGGCA |  |  |  |
| *Mac-1* | CCATGACCTTCCAAGAGAATGC | ACCGGCTTGTGCTGTAGTC |  |  |  |
| *Irf8* | AGACGAGGTTACGCTGTGC | TCGGGGACAATTCGGTAAACT |  |  |  |
| *Ltf* | TGAGGCCCTTGGACTCTGT | ACCCACTTTTCTCATCTCGTTC |  |  |  |
| *Itgax* | CTGGATAGCCTTTCTTCTGCTG | GCACACTGTGTCCGAACTCA |  |  |  |
| *Cdkn1c* | CGAGGAGCAGGACGAGAATC | GAAGAAGTCGTTCGCATTGGC |  |  |  |
| *Cdkn2c* | CCTTGGGGGAACGAGTTGG | AAATTGGGATTAGCACCTCTGAG |  |  |  |
| *Cdkn1b* | TCAAACGTGAGAGTGTCTAACG | CCGGGCCGAAGAGATTTCTG |  |  |  |
| *Meis1* | GCAAAGTATGCCAGGGGAGTA | TCCTGTGTTAAGAACCGAGGG |  |  |  |
| *Hoxa9* | CCCCGACTTCAGTCCTTGC | GATGCACGTAGGGGTGGTG |  |  |  |
| *Ccne2* | ATGTCAAGACGCAGCCGTTTA | GCTGATTCCTCCAGACAGTACA |  |  |  |
| *Il3ra* | CTGGCATCCCACTCTTCAGAT | GGTCCCAGCTCAGTGTGTA |  |  |  |
| *Ccnd1* | GCGTACCCTGACACCAATCTC | CTCCTCTTCGCACTTCTGCTC |  |  |  |
| *Pbx3* | CGAGGCGCAAGCAAAGAAAC | TGCCAAAAGCATATTGTCCAGT |  |  |  |
| *Gapdh* | AGGTCGGTGTGAACGGATTTG | TGTAGACCATGTAGTTGAGGTCA |  |  |  |
| *Mpo* | AGTTGTGCTGAGCTGTATGGA | CGGCTGCTTGAAGTAAAACAGG |  |  |  |
| *Mbd2* | AGAACAAGGGTAAACCAGACCT | ACTTCACCTTATTGCTCGGGT |  |  |  |
| *Mbd1* | AAACGCCGAGAGTCCTTTCG | GGTAATAGATGTCTGAGCGTCCA |  |  |  |
| *Mbd3* | CCCCAGCGGGAAGAAGTTC | CGGAAGTCGAAGGTGCTGAG |  |  |  |
| *Mbd4* | GGACAACAGAGTCCGTGGAG | ATCACCAGGTCCTTTCCATCT |  |  |  |
| *Mecp2* | ATGGTAGCTGGGATGTTAGGG | TGAGCTTTCTGATGTTTCTGCTT |  |  |  |
| **Human** | | |  |  |  |
| *CDKN1A* | TGTCCGTCAGAACCCATGC | AAAGTCGAAGTTCCATCGCTC |  |  |  |
| *CDKN1C* | GCGGCGATCAAGAAGCTGT | GCTTGGCGAAGAAATCGGAGA |  |  |  |
| *CDKN2C* | GGGGACCTAGAGCAACTTACT | CAGCGCAGTCCTTCCAAAT |  |  |  |
| *CDKN1B* | TAATTGGGGCTCCGGCTAACT | TGCAGGTCGCTTCCTTATTCC |  |  |  |
| *GAPDH* | GACAGTCAGCCGCATCTTCT | TTAAAAGCAGCCCTGGTGAC |  |  |  |
| *MBD2* | ATACAGAAGAGATGGATATTGAA | GGCATTGGTTAGTGCTATT |  |  |  |

**Table S2. The sequence of primers used in ChIP assays**

|  | Forward Primer (5'-3') | Reverse Primer (5'-3') |
| --- | --- | --- |
| *GAPDH* | AACTAGGATGGTGTGGCTCC | ATCGCCCCACTTGATTTTGG |
| *MAFB* | CACTCTCCTCCCTCAGTTCC | GCGCAGATCTCCAGAAACAG |
| *CDKN1C-1* | GCGCCAGCTGACGAGGCT | AAGAGTGGAGCTGACCCCTGTC |
| *CDKN1C-2* | CGCACTAGTACTGGGAAGGT | TTTCCCCTTCTTCTCGCTGT |

**SUPPLEMENTAL FIGURES AND SUPPLEMENTAL FIGURE LEGENDS**


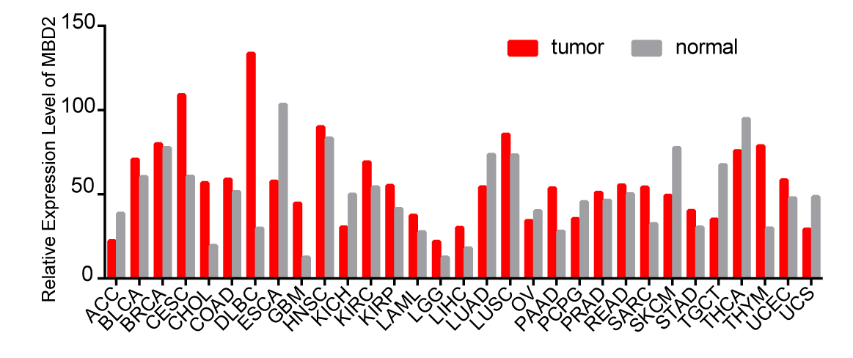


**Supplementary Fig. S1. The potential clinical relevance of MBD2 expression levels in the 31 different types of cancers.** Box plots showing MBD2 mRNA expression in tumors (red plot) and their corresponding normal (grey plot) tissues using data from the TCGA database through GEPIA. Compared with their corresponding normal tissues, MBD2 is over-expressed in the 21 types of cancers and down-regulated in the remaining 10 types. ACC, Adrenocortical carcinoma; BLCA, Bladder urothelial carcinoma; BRCA, Breast invasive carcinoma; CESC, Cervical squamous cell carcinoma and endocervical adenocarcinoma; CHOL, Cholangiocarcinoma; COAD, Colon adenocarcinoma; DLBC, Diffuse large B-cell lymphoma; ESCA, Esophageal carcinoma; GBM, Glioblastoma multiforme; HNSC, Head and neck squamous cell carcinoma; KICH, Kidney chromophobe; KIRC, Kidney renal clear cell carcinoma; KIRP, Kidney renal papillary cell carcinoma; LAML, Acute myeloid leukemia; LGG, Lower grade glioma; LIHC, Liver hepatocellular carcinoma; LUNG, Lung cancer; LUAD, Lung adenocarcinoma; LUSC, Lung squamous cell carcinoma; OV, Ovarian serous cystadenocarcinoma; PAAD, Pancreatic adenocarcinoma; PCPG, Pheochromocytoma and paraganglioma; PRAD, Prostate adenocarcinoma; READ, Rectum adenocarcinoma; SARC, Sarcoma; SKCM, Skin cutaneous melanoma; STAD, Stomach adenocarcinoma; TGCT, Testicular germ cell tumor; THCA, Thyroid carcinoma; THYM, Thymoma; UCEC, Uterine corpus endometrial carcinoma; UCS, Uterine carcinosarcoma.


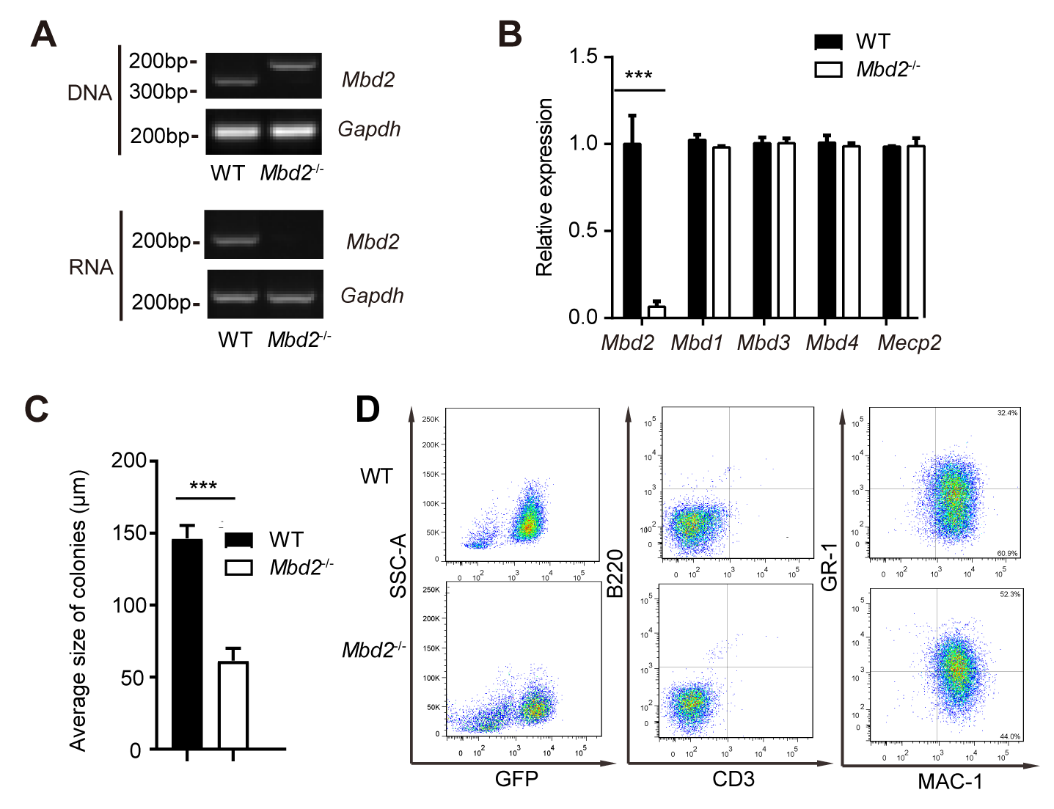


**Supplementary Fig. S2. MBD2 contributed to the initiation of AML in an MLL-AF9-driven mouse model.**

**A** BM was obtained from WT and *Mbd2*^-/-^ mice. Upper panel, genomic DNA was isolated and amplified by PCR to confirm the deletion of *Mbd2*. Lower, the expression of *Mbd2* transcripts was determined by *q*RT-PCR in the depicted cells. *Gapdh* was included as a loading control. **B** The expression levels of other members of the MBD family transcripts (*Mbd1, Mbd3, Mbd4* and *Mecp2*) were determined by *q*RT-PCR in the indicated cells. *Gapdh* was included as a loading control. Error bars, s.d. **C** Average size of colonies from either *Mbd2*^-/-^ or WT mice on day 21. Error bars, s.d. **D** Immunophenotypic features of colonies from either *Mbd2*^-/-^ or WT mice. GFP^+^ cells were gated (left panel) and assessed for lymphoid (middle panel) and myeloid markers (right panel). ****P* < 0.001.

**
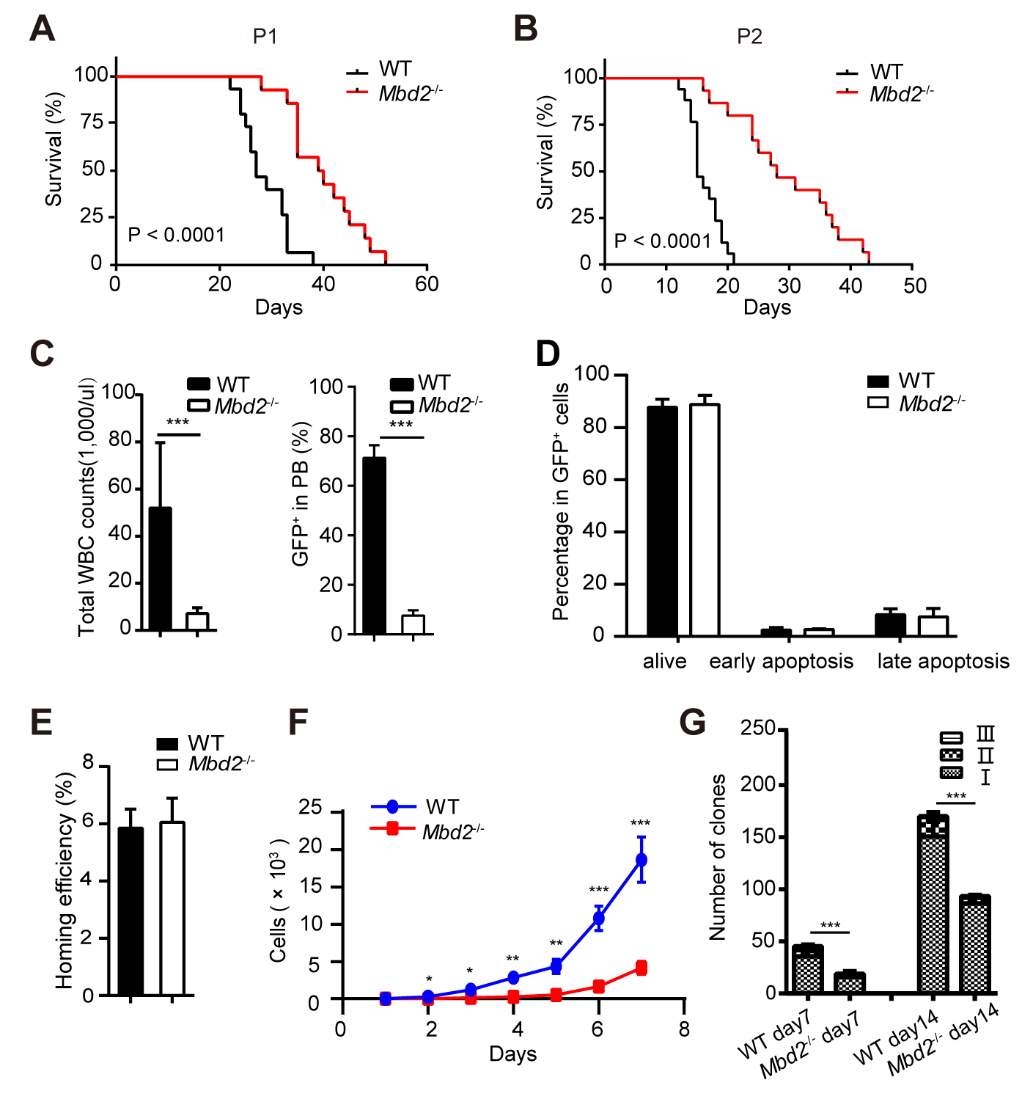
**

**Supplementary Fig. S3. Loss of MBD2 significantly attenuated the development of MLL-rearranged leukemia.**

**A-B** Kaplan-Meier survival curves for P1 (A) and P2 (B) recipient mice transplanted with GFP^+^ leukemic cells. **C** WBCs (left) and the percentage of GFP^+^ cells (right) in the PB of mice at 4 weeks that received transplants of *Mbd2*^-/-^ and WT leukemia cells (n = 6). WBC, White blood cell; PB, Peripheral blood. Error bars, s.d. **D** Bars indicate the results of apoptosis assays in WT or *Mbd2*^-/-^ AML cells using 7-AAD and annexin V. Error bars, s.d. **E** Homing assay. Bars indicate the frequency of homing CFSE^+^ mononuclear cells found in the BM of recipient mice. Error bars, s.d. **F** Liquid culture was performed *in vitro* to investigate the dynamic effect of GFP^+^ AML cells from *Mbd2*^-/-^ and WT mice*.* Error bars, s.d. **G** Bar graphs show the colony-forming capacity of *Mbd2*^-/-^ AML cells compared to that of WT leukemia cells. Colonies were enumerated every 7 days in semisolid culture. Error bars, s.d. **P* < 0.05, ***P* < 0.005, and ****P* < 0.001.


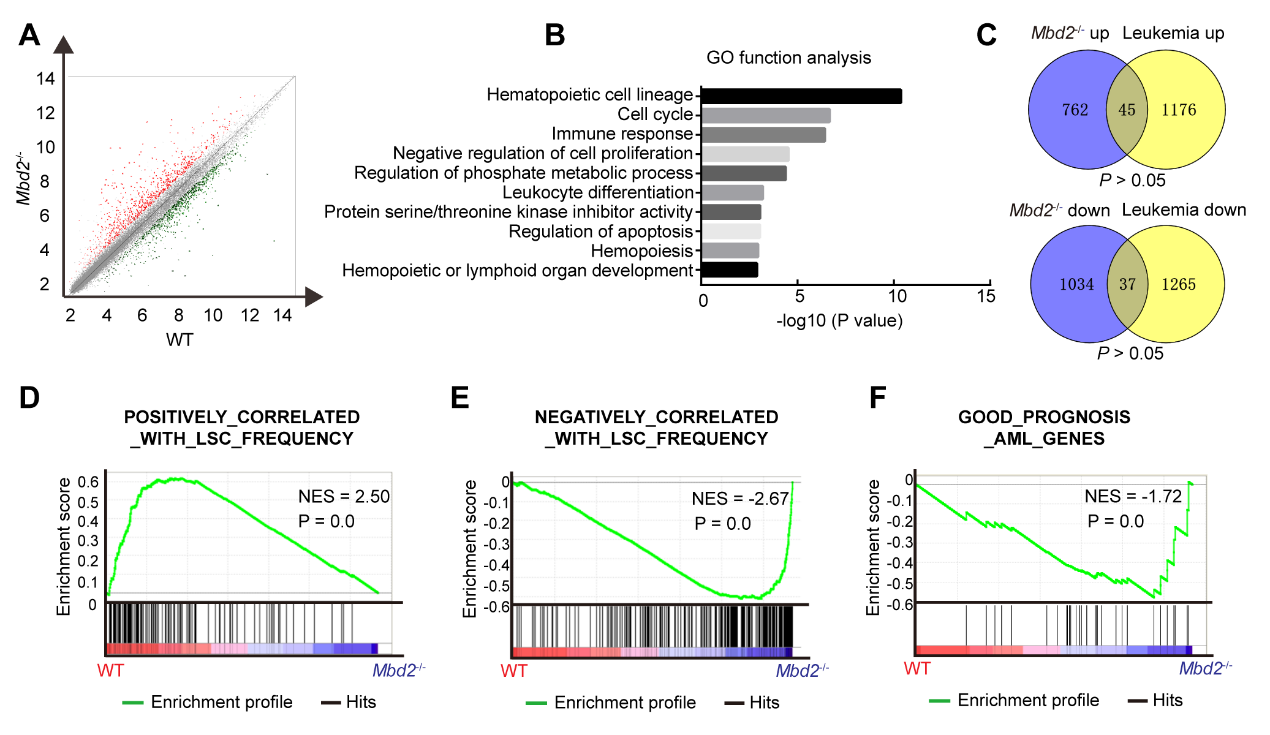


**Supplementary Fig. S4. MBD2 altered the expression profile of MLL-AF9 leukemia and led to down-regulation of the expression of LSC genes.**

**A** Scatter plots of the expression profiles from the WT and *Mbd2*^-/-^ MLL-AF9 groups. **B** DAVID enrichment analyses were performed using the Gene Ontology database. Data are shown as -log10 (*P* value). **C** Venn diagrams displaying the intersection of the same trend DEGs in WT and *Mbd2*^-/-^ AML cells from our microarray data (blue circle, ‘*Mbd2*^-/-^ up’ and ‘*Mbd2*^-/-^ down’ represent the upregulated or downregulated genes in *Mbd2*^-/-^ leukemic cells, respectively), and DEGs in leukemia and normal BM from the GSE34185 dataset (yellow circle, ‘leukemia up’ and ‘leukemia down’ represent the upregulated or downregulated genes in AML cells, respectively). Both *P* > 0.05, calculated using the R package SAGx_1.32.0 under R version 2.15.3. **D-F** GSEA plot showing down-regulated genes in the *Mbd2*^-/-^ AML groups that are positively correlated with LSC self-renewal (D), upregulated genes in the *Mbd2*^-/-^ AML groups that are negatively correlated with LSC self-renewal (E), and upregulated genes that are associated with good prognosis in AML (F).


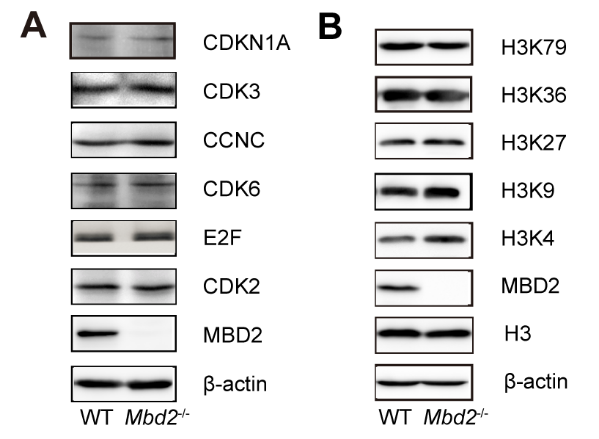


**Supplementary Fig. S5. Loss of MBD2 induced cell cycle arrest in LSCs by activating the transcription of cell cycle inhibitors.**

**A** WB analysis showed that CDKN1A, CDK3, CCNC, CDK6, E2F, and CDK2 were also unaffected by MBD2 deletion; β-actin was used as the loading control. **B** WB analysis of a panel of specific histone lysine and arginine methylation sites in *Mbd2*^-/-^ and WT MLL-AF9 leukemia cells; β-actin was used as the loading control.

**
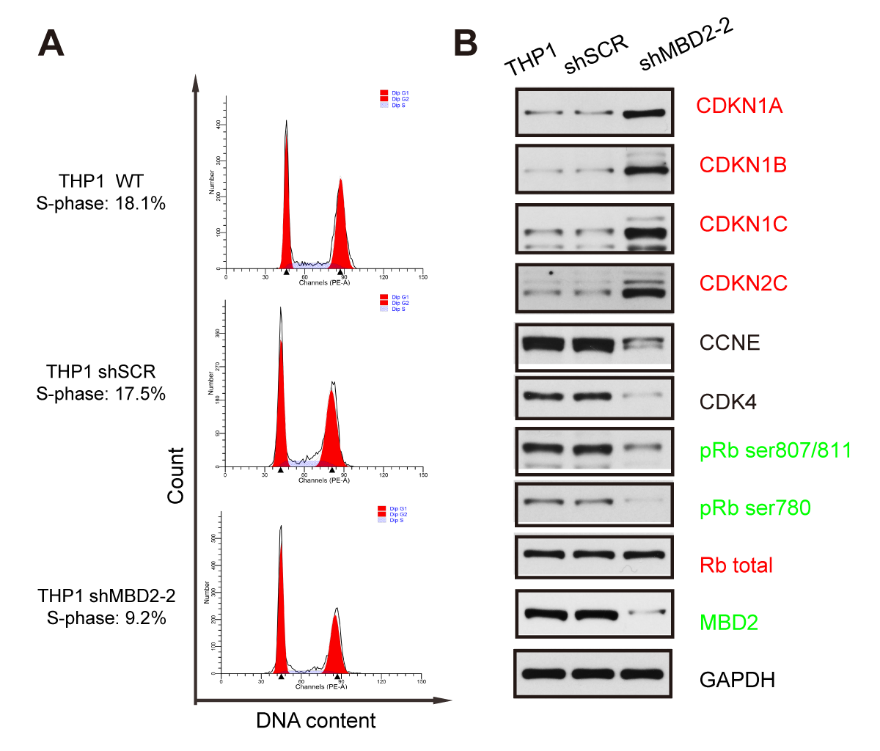
**

**Supplementary Fig. S6.** **MBD2 knockdown decreased THP1 cell growth by shMBD2-2.**

**A** Typical flow cytometric profiles show representative cell cycle data for parental THP1, shSCR and shMBD2-2 cells. The cell cycle was analyzed using PI staining. **B** Expression levels of typical cell cycle regulators in parental THP1, shSCR and shMBD2-2 cells were determined by WB. GAPDH was used as internal control.

**
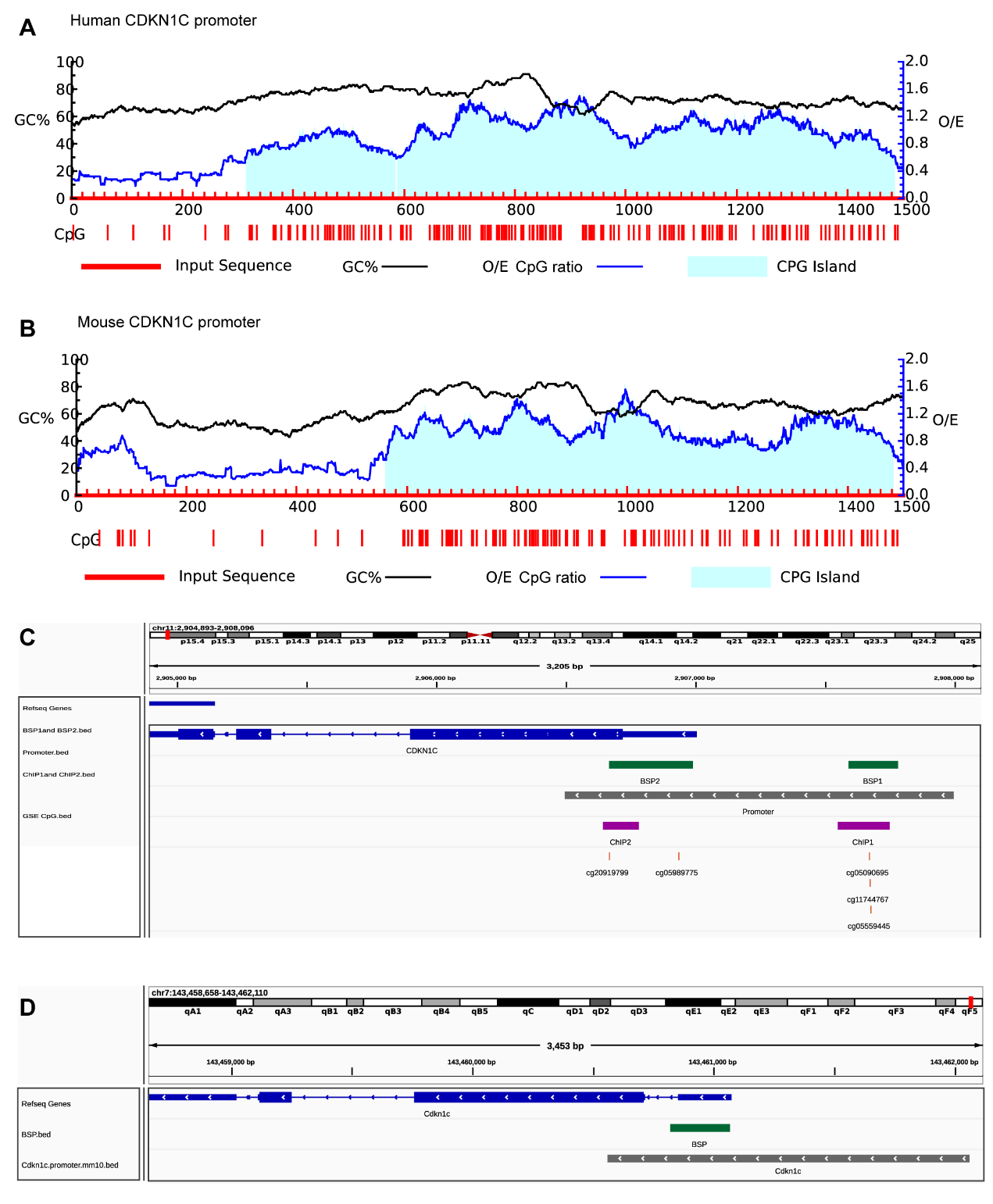
**

**Supplementary Fig. S7.** **Schematic diagram of the CpG island and the precise position of BSP and ChIP in the promoter region of CDKN1C.**

**A-B** MethPrimer 2.0 showed the prediction of the CpG island in the promoter region of CDKN1C in humans (A) and mice (B). Vertical bars indicate CpG dinucleotides. **C-D** Integrative Genomics Viewer showed that the precise position of CDKN1C promoter for bisulfite sequencing PCR (BSP) and chromatin immunoprecipitation (ChIP) in humans (C) and mice (D), respectively. BSP1 position, 191 bp, 2907589-2907779 (hg19); BSP2 position, 325 bp, 2906667-2906991(hg19). ChIP1 position, 200 bp, 2907550-2907749 (hg19); ChIP2 position, 138 bp, 2906643-2906780 (hg19). Mouse BSP position, 249 bp, 143460819-143461067 (mm10).


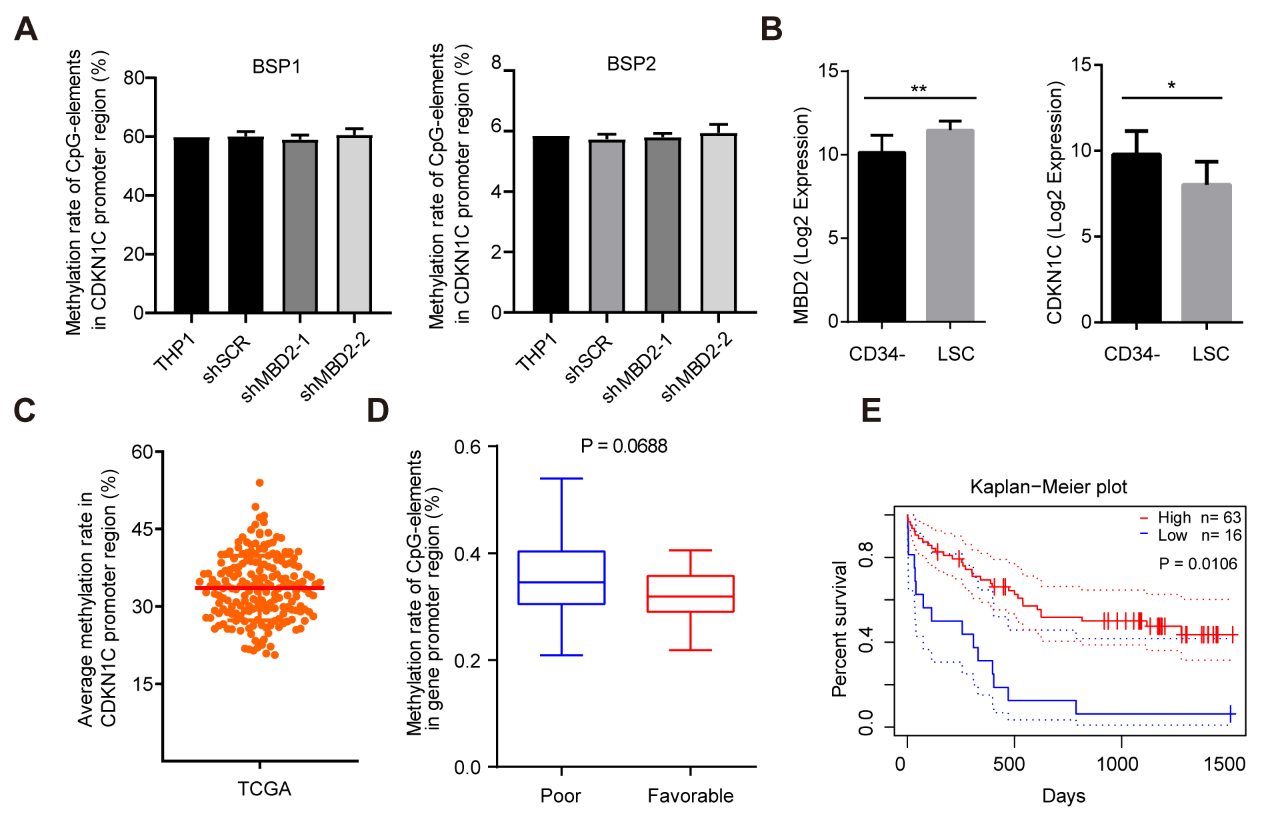


**Supplementary Fig. S8. Low expression levels and high methylation levels of CDKN1C indicate a poor prognosis in AML.**

**A** The CDKN1C methylation levels of the BSP1 and BSP2 regions for parental THP1, THP1 shSCR, shMBD2-1 and shMBD2-2 cells are shown. Bisulphite sequencing PCR, BSP. Error bars, s.d. **B** Analysis of MBD2 (left) and *CDKN1C* (right) expression levels in LSCs and CD34^-^ cells from AML patients in GSE24006. Error bars, s.d. **C** Average CpG methylation levels of the *CDKN1C* promoter region in AML patients from TCGA database. **D** The average methylation levels of *CDKN1C* in AML patients with a poor prognosis versus AML patients with a favorable prognosis from the TCGA database. Error bars, s.d. **E** Survival curves comparing AML patients with high (red) and low (blue) expression of *CDKN1C* in the GSE12417 dataset [11] were plotted from the PrognoScan database. Patients were divided into a *CDKN1C*-high group (n=63) and a *CDKN1C*-low group (n =16) according to the median *CDKN1C* expression level (219533_at) in the GSE12417 dataset. **P* < 0.05, ***P* < 0.005.
